# Heat Shock Protein 90 is overexpressed in high-risk myelodysplastic syndromes and associated with higher expression and activation of Focal Adhesion Kinase

**DOI:** 10.18632/oncotarget.557

**Published:** 2012-09-28

**Authors:** Pascale Flandrin-Gresta, Françoise Solly, Carmen Mariana Aanei, Jérôme Cornillon, Emmanuelle Tavernier, Nathalie Nadal, Franck Morteux, Denis Guyotat, Eric Wattel, Lydia Campos

**Affiliations:** ^1^ Laboratoire d'Hématologie, University Hospital of Saint-Etienne, University Hospital of Saint-Etienne, 42055 Saint-Etienne Cedex 2, France; ^2^ UMR5239 CNRS, Université Claude Bernard Lyon 1, Faculté deMédecine J Lisfranc Saint-Etienne, 42023 Saint-Etienne Cedex 2, France; ^3^ Institut de Cancérologie de la Loire, University Hospital of Saint-Etienne, 42271 Saint Priesten Jarez cedex, France

**Keywords:** HSP90, MDS, FAK, 17-AAG

## Abstract

Myelodysplastic syndromes are characterized by a high risk of evolution into acute myeloid leukaemia which can involve activation of signalling pathways. As the chaperone heat shock protein 90 (HSP90) has a key role in signal transduction, we investigated its role in the pathogenesis and evolution of myelodysplastic syndromes. Expressions of HSP90 and signalling proteins clients (phosphorylated-AKT (pAKT), Focal Adhesion Kinase (FAK) and phosphorylated-FAK (pFAK)), were assessed in bone marrow mononuclear and CD34-positive (CD34^+^) cells from 177 patients with myelodysplasia. Effects of HSP90 inhibition were also evaluated in 39 samples. The levels of all proteins studied were significantly higher in patients with high grade disease, than those with low grade myelodysplastic syndrome or chronic myelomonocytic leukaemia. High levels of HSP90, FAK, pFAK and pAKT were associated with shorter survival and increased risk of progression into acute leukaemia. A down regulation of pFAK and pAKT and increased apoptosis was observed in mononuclear and CD34^+^ cells after 12 hours of incubation with 17-AAG. In conclusion, our data suggest the implication of HSP90 and FAK and AKT activation in the pathogenesis of myelodysplastic syndromes with excess of blasts and evolution to leukaemia. Moreover this signalling network could be a therapeutic target through HSP90 inhibition.

## INTRODUCTION

The myelodysplastic syndromes (MDS) are a heterogeneous group of diseases with regard to initial presentation and evolution [1]. Patients with MDS usually present with one or several peripheral cytopenias despite a normo- or hypercellular bone marrow (BM). This apparent paradox has been linked to an excessive intramedullary apoptosis [2-4], but mechanisms underlying this phenomenon are not fully understood yet. We and others have demonstrated that apoptosis results from the activation of caspases, particularly caspase 3 [5,6]. The microenvironment is also implicated in the pathogenesis through the secretion of proapoptotic cytokines (Fas and Trail) [7,8]. Increased apoptosis is observed in all forms of MDS, but is higher in patients with better prognosis and comparatively lower in patients with an excess of blasts [5,6].

Apoptosis is a tightly regulated phenomenon, and caspase activation is controlled by the bcl-2-family proteins. Indeed we showed that the apoptotic disorder was associated with an imbalance between proteins of the bcl-2 family, with an upregulation of anti-apoptotic proteins bcl-2 and bcl-X_L_ in the forms with excess of blasts [9]. More recently the role of Heat Shock Proteins (HSP) in cell protection and apoptosis regulation has been demonstrated. HSP are a group of highly conserved proteins, which act as molecular chaperones in order to ensure the proper folding of synthesized proteins, or their refolding under denaturating conditions [10]. They also play a role in protein degradation via the proteasome machinery. A member of the HSP family, HSP90, is abundantly expressed in the cytoplasm of most human cells. HSP90 exists in two main isoforms: HSP90α, inducible, and HSP90β, constitutive [11]. It exerts its role by forming a multiprotein complex with high ATPase activity, in cooperation with cochaperones, including HSP70 [12]. HSP90 clients are implicated in cell cycling, receptor function, signal transduction and apoptosis. High levels of HSP90α protein or HSP90 mRNA have been reported in many types of cancer cells, such as pancreatic carcinomas, breast cancer, ovarian cancer, lung and renal cancer, gastric cancer (reviewed by Ochel *et al*) [13]. Furthermore, HSP90 exists mainly in the activated (complexed) form in cancer cells, whereas in non malignant cells only a small part of HSP90 is activated [14].

More limited data are available regarding the expression of HSP90 in haematological malignancies and particularly in acute leukaemia and MDS: high expression of HSP90 protein and HSP90α RNA has been reported by Yufu *et al* in leukemic cell lines and a small series of acute leukaemia patients [15]. We reported on the expression of HSP90 in a larger series of patients with acute myeloid leukaemia (AML) [16]. Higher HSP90 levels, as assessed by flow cytometry, were associated with a poor prognosis and higher expression of activated signal transduction proteins: phosphoinositide 3-kinase (PI3K), phospho serine-threonine protein kinase AKT (also known as protein kinase B) and extracellular signal-regulated kinases (ERK). Other reports show that HSP90 is necessary for the maintenance of oncoproteins such as bcr-abl [17], mutated c-kit [18], and flt3 [19,20].

HSP90 activation and functional properties necessitate the binding of ATP to a specific pocket. The benzoquinone ansamycins herbimycin A and geldanamycin are potent inhibitors of HSP90, binding tightly to the ATP pocket and preventing the formation of an active HSP90 complex [21]. The less toxic geldanamycin-derivative 17-allylamino-demethoxy geldanamycin (17-AAG) presents a much higher (up to 100-fold) affinity for HSP90 complexes than for uncomplexed HSP90, which confers to this drug a highly specific anti-tumoral activity [22]. 17-AAG (Tanespimycin) and other HSP90 inhibitors are now considered as targeted therapy for cancer, as they show promise in early clinical trials [23,24]. In a preliminary study, we have shown that HSP27, 70 and 90 are over-expressed in advanced MDS as compared to early MDS and normal BM [25]. This suggests their possible implication in MDS pathogenesis and evolution. Here we report on the clinical and biological significance of HSP90 expression in a series of 177 patients with MDS. We evaluated the expression of HSP90 and of relevant client proteins (pAKT), implicated in cell survival and autonomous growth, and phospho-focal adhesion kinase (pFAK), implicated in tissue invasion and metastasis, at diagnosis and in some cases after evolution to a higher grade MDS or to overt AML. The use of multicolour flow cytometry allowed us to specifically study subsets of cells (ie CD34+ cells). We show that HSP90 and FAK are overexpressed in high risk cases, and that CD34+ cells are highly sensitive to the HSP90 inhibitor 17-AAG.

## RESULTS

### Expression of HSP90, FAK, pFAK and pAKT

Expression of HSP90 was weak in normal bone marrow MNC (MFIR: 7.8 ± 2.3, n= 6). We also observed a low expression of FAK and pFAK (MFIR : 4.2 ± 0.8 ; 6.8 ± 0.8 respectively), whereas pAKT was not detected above control level. Results were similar in normal CD34+ cells for all the proteins studied.

**Figure 1:**
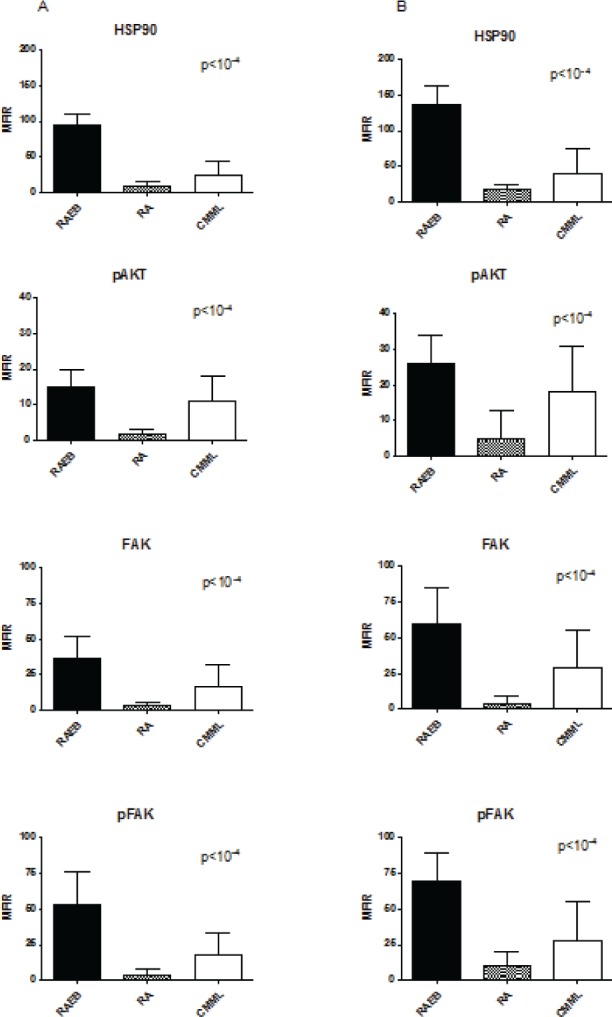
Level of HSP90, pAKT, FAK and pFAK expression according to MDS subgroups The mean fluorescence intensity ratio (MFIR) for each protein in bone marrow MNC (A) or CD34+ cells (B) was evaluated in high risk MDS patients (RAEB, n=93) versus low risk MDS patients (RA, comprising the RAUD, RARS, RCMD, RCMD-RS and 5q- subgroups, n=61), and CMML patients (n=23).

In MDS/CMML MNC, HSP90 and other proteins levels were significantly higher in high-risk cases according to WHO classification (p<10^−4^, Figure 1, mean MFIR ± SD in refractory anaemia with excess of blasts RAEB (n=93) versus refractory anaemia RA (n=61) and CMML (n=23), respectively: 37 ± 21 versus 7 ± 4 and 22 ± 21 for HSP90, 26 ± 17 versus 6± 5 and 16 ± 16 for AKT, 33 ± 18 versus 5 ± 6 and 19 ± 23 for FAK, 31 ± 18 versus 3 ± 5 and 15 ± 16 for pFAK). The expression of HSP90 was also higher in RAEB-II versus RAEB-I (p < 0.05) and there was a trend for higher levels in refractory cytopenia with multilineage dysplasia (RCMD) versus RA with or without ringed sideroblasts (p=0.06). Similar results were obtained when considering the percentage of positive cells instead of the MFIR (data not presented).

Although these proteins were not detected only in the “blast” gate (CD45/SSC low) of MNC, we observed a weak linear correlation between the percentage of blasts (as assessed by cytology or by cytometry) and the expression of HSP90, pAKT, FAK and pFAK in MNC (r^2^=0.62 to 0.71).

These proteins were also expressed at significantly higher levels in CD34+ cells than in CD34-negative MNC (p<10^−4^ for all proteins). We therefore compared the level of expression in CD34+ cells in the different sub-types of MDS and in CMML. Again, we observed a higher expression of HSP90 (mean MFIR ± SD: 62 ± 22 versus 5 ± 6 and 25 ± 24), pAKT (51 ± 22 versus 4 ± 5 and 17 ± 22), FAK (37 ± 13 versus 4 ± 4 and 17 ± 21) and pFAK (69 ± 20 versus 4 ± 6 and 22 ± 27) in RAEB (n=93) than in RA (n=61), and CMML (n=23) respectively, CMML exhibiting intermediate levels (Figure 1). This shows that the differences regarding expression in MNC were not only due to the higher percentage of blasts or CD34+ cells in high-risk cases.

**Figure 2:**
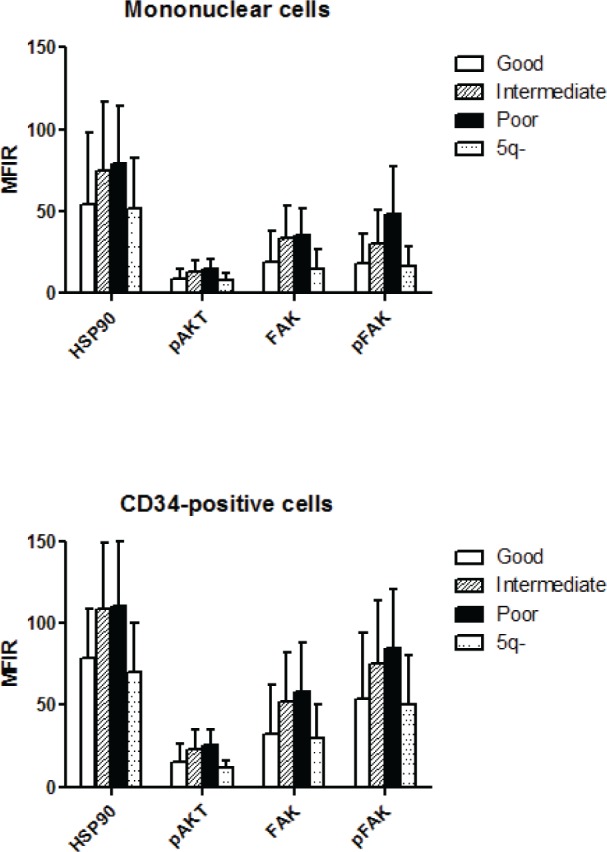
Level of HSP90, pAKT, FAK and pFAK expression according to cytogenetic subgroup The mean fluorescence intensity ratio (MFIR) for each protein in bone marrow MNC or CD34+ cells was evaluated in MDS patients with good (n=91), intermediate (n=32) or poor (n=29) prognosis caryotypes, and in patients with 5q- syndrome (n=14)

Finally, there were also significant differences related to cytogenetic category (overall p level <10^−4^, figure 2). Specifically, HSP90 (p<0.001), FAK (p<0.05), and pFAK (p<0.01) expression in MNC was lower in good prognosis than in intermediate/poor prognosis forms. pFAK levels were more elevated in poor prognosis than in intermediate forms (p<0.05). The same differences were observed in CD34+ cells, but in addition, pAKT levels were lower in good versus intermediate or poor prognosis groups (p<10^−4^). 5q- cases expressed similar levels to other good prognosis cases.

### Predictive value

**Figure 3:**
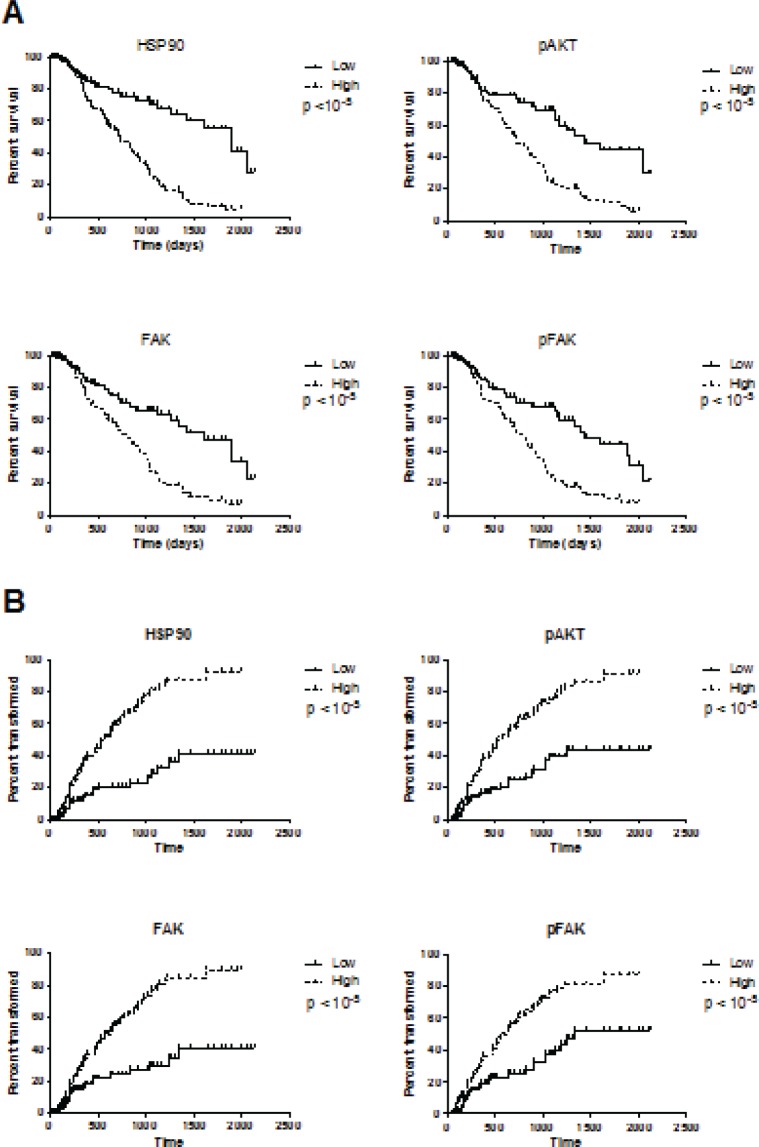
Survival duration (A) and time to transformation into overt leukemia (B) according to the levels of HSP90, pAKT, FAK and pFAK expression Patients were separated into two categories: those with a high level of protein expression when the MFIR for this protein was equal to or above the median value of the entire cohort, or low level of protein expression when the MFIR was below the median value.

Because of the correlation with other relevant clinical factors such as cytogenetic and percentage of blasts, we studied the prognostic value of HSP90, pAKT, FAK and pFAK expression. For univariate analysis, patients were arbitrarily placed into two categories: high level (MFIR equal to or above median value) or low level (MFIR below median value). Survival duration and time to transformation into overt leukaemia were significantly shorter in patients with high expression of HSP90, pAKT, pFAK and FAK (Figure 3). For all those intracellular proteins, the percentage of positive cells was significantly higher in the groups with increasingly poor prognosis as defined by the international prognostic scoring system IPSS (p<10^−4^, Table 2). In multivariate analysis, we studied the effects of known parameters such as age, cytogenetic (or IPSS), percentage of blasts and marker expression. Only cytogenetics (or IPSS), percentage of blasts and percentage of CD34+ cells remained independent prognostic factors.

**Table 1:** Patients characteristics

Median age (range)	66 years (11-91)
Sex (M/F)	99/78
WHO classification (N)
RCUD	20
RARS	1
RCMD	24
RAEB-I	38
RAEB-II	55
5q-	14
MDSu	2
CMML (N)	23
Cytogenetic prognostic groups (N=152)
Good	91
Intermediate	32
Poor	29
IPSS (N=152)	
Low	56
Intermediate-1	44
Intermediate-2	32
High	20
Median follow-up (days)	593
Median time to transformation (days) (N=87)	386

RAUD: refractory anemia with unilineage dysplasia, RARS: refractory anemia with ring sideroblasts, RCMD: refractory anemia with multilineage dysplasia, RAEB-I: refractory anemia with excess of blasts type 1, RAEB-II: refractory anemia with excess of blasts type 2, 5q-: deletion 5q syndrome, MDSu: myelodysplastic syndrome, unclassifiable, RA: refractory anemia, CMML: chronic myelo-monocytic leukemia, IPSS: international prognostic score system.

**Table 2:** Expression of HSP90, pAKT, FAK and pFAK in CD34+ bone marrow cells according to IPSS risk category (p<10-4)

		Mean Fluorescence Intensity Ratio (SD)			
IPSS	N	HSP90	pAKT	FAK	pFAK
Low	34	13 (12)	6 (4)	5 (5)	4 (4)
Int-1	51	73 (61)	9 (7)	21 (20)	29 (23)
Int-2	42	85 (64)	13 (11)	29 (24)	47 (40)
High	25	86 (69)	35 (12)	34 (29)	51 (45)

### Transformation into AML

Transformation occurred in 87 cases in total, after a mean delay of 386 days. Nineteen cases could be reevaluated at the time of transformation. This included 9 cases with a diagnosis of RA and 10 RAEB. The blast percentage was 6.5% (±5.9) at diagnosis and 42% (±16) after transformation.

**Figure 4:**
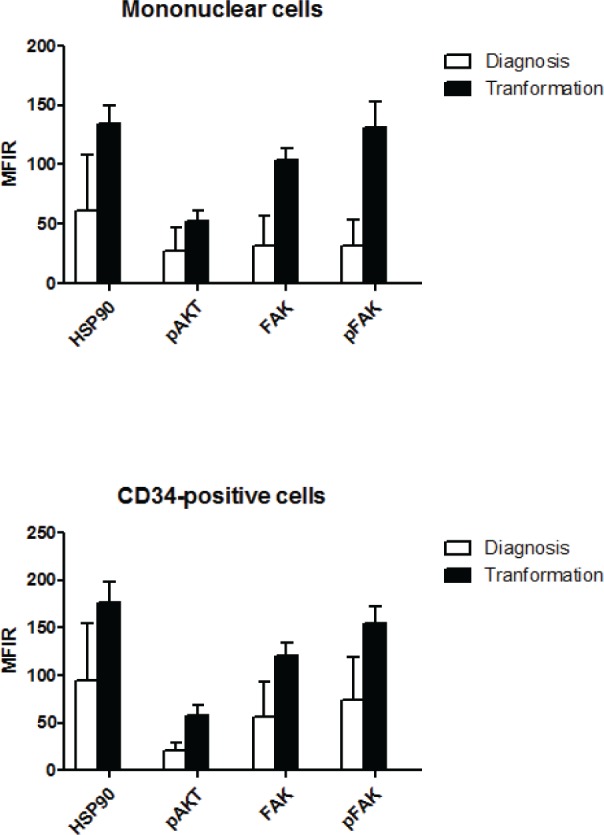
Comparative levels of HSP90, pAKT, FAK and pFAK expression at diagnosis and after transformation into acute leukemia, in MNC and CD34+ cells Nineteen patients could be evaluated, 9 with an initial diagnosis of RA and 10 with an initial diagnosis of RAEB.

As presented in Figure 4, the levels of HSP90, FAK, pFAK and pAKT were significantly higher after transformation than at diagnosis. This was the case in MNC (p<10^−4^) and more interestingly in CD34+ cells (p<10^−4^).

### Inhibition of HSP90

**Figure 5:**
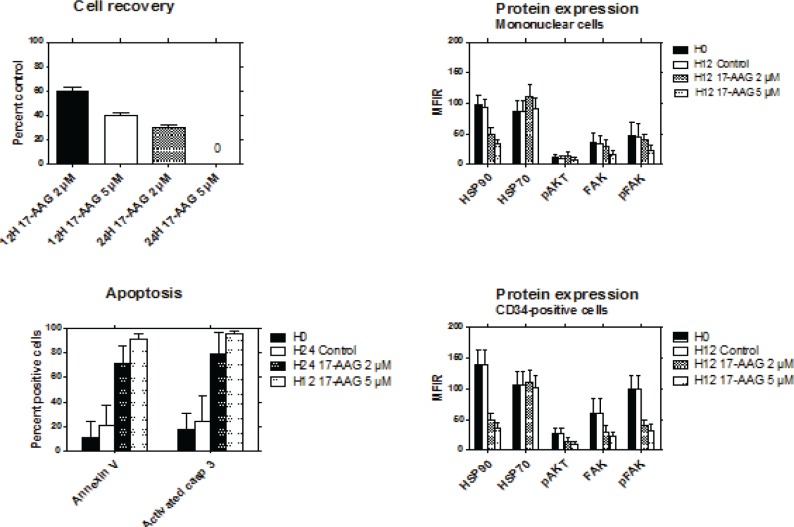
Effects of HSP90 inhibition with 17-AAG The viability of bone marrow MNC from RAEB patients (n=39) was studied after exposure to 2μM or 5μM of 17-AAG for 12 to 24 hours (top left). Apoptosis of the cells was assessed by activated caspase-3 expression and annexin V binding after exposure to 2μM or 5μM of 17-AAG for 24 hours and 12 hours respectively (bottom left). The levels of HSP90, HSP70, pAKT, FAK and pFAK expression was evaluated in MNC (top right) and CD34+ cells (bottom right) after exposure to 2μM or 5μM of 17-AAG for 12 hours.

The effects of inhibition of HSP90 were studied by exposing MNC to 17-AAG for up to 24 hours. Thirty-nine cases of RAEB expressing high levels of HSP90 were studied. In the presence of 2 μM 17-AAG, the percentages of viable cells at 12 and 24 hours were respectively 60% and 32% compared to 100% for the control without 17-AAG. At a concentration of 5 μM, the percentage of viable cells was 38% at 12 hours, and no viable cell could be recovered at 24 hours (Figure 5).

17-AAG significantly increased the percentage of apoptotic cells, as assessed by activated caspase-3 expression and annexin V binding. After 12 hours of exposure to 5 μM 17-AAG, more than 90% of cells were apoptotic (Figure 5). In clonogeneic assays, the yield of colonies was heterogeneous in the absence of 17-AAG (mean number ± SD of CFU-GM, clusters and BFU-E: 60 ± 80, 139 ± 136, and 19 ± 14, respectively). At a concentration of 5 μM, 17-AAG completely inhibited the growth of all types of colonies in semi-solid medium (data not shown).

The effects of 17-AAG on protein expression was studied after 12 hours of culture. The same staining technique was used as for uncultured cells. In addition, we also assessed the expression of HSP70, which has been reported to be upregulated in the presence of HSP90 inhibitors. The levels of HSP90, FAK, pFAK and pAKT were significantly decreased in the presence of 2 and 5 μM of 17-AAG. This effect was more evident in CD34+ cells than in MNC. By contrast, expression of HSP70 was not significantly modified in both cell types (Figure 5).

## DISCUSSION

In this paper, we show that high expression of HSP90 is associated with increasingly advanced disease and poor clinical prognosis. As expected, this was significantly correlated with other known prognostic features such as cytogenetics and blast percentage which appear to be of importance in multivariate analysis. One interesting point is the fact that CD34+ cells from advanced cases also expressed the higher levels, when compared to CD34+ cells of good prognosis cases or normal marrows. This implies that progression to advanced disease is associated with a distinct transduction pathways profile at the CD34+ progenitor level, where an imbalance between proliferation and apoptosis has already been described.^6^ In line with this observation, we also show that when patients could be retested at the time of transformation into overt leukemia, the expression of HSP90 and signaling molecules was significantly increased. This is also consistent with the known overexpression of HSP90 in acute myeloid leukemia [15, 16].

We also studied the expression of the activated forms of known clients of HSP90, pAKT and pFAK. These proteins are implicated in signal transduction and their constitutive activation is a hallmark of transformation in many cancer models. Again a higher expression was observed in MNC and in CD34+ cells of MDS with poor prognosis or adverse cytogenetics. Indeed high expression of HSP, FAK and pAKT was associated with higher risk of transformation and poor survival. High levels of HSP and FAK are predictive of resistance to chemotherapy in AML, as shown by our group and others [16, 30]. The data regarding the prognostic significance of pAKT activation in AML cells is more controversial. While we and others have observed a correlation with poor prognosis [16, 29], Tamburini *et al* showed that pAKT expression as detected by flow cytometry implied a better prognosis, observed independently of cytogenetics, although pAKT was higher in core binding factor (CBF) and intermediate cases [31]. These discrepancies may be the consequence of different technical approaches, but mainly due to the fact that different sites of phosphorylation were assessed in these studies. Again, we show that levels in CD34+ cells were higher at time of evolution to AML than at diagnosis, irrespective of the initial classification of MDS. Taken together, these findings favor a role for these proteins in disease initial type and in the pathogenesis of evolution into overt AML.

The second point raised in our study is the possibility to target *in vitro* HSP90. HSP90 inhibitors such as geldanamycin, 17-AAG or 17-DMAG have anti-tumoral activity by inhibiting proliferation and inducing apoptosis [32, 33]. In leukemia cells, 17-AAG alone or in combination with chemotherapy inhibits cell growth and induces apoptosis [34]. Moreover, in specific leukemia subtypes characterized by the presence of mutations or rearrangements of genes such as *FLT-3* or *BCR-ABL* resulting in high expression of constitutively activated oncoproteins, the association of HSP90 inhibitors and targeted drugs is highly effective *in vitro* [35, 36]. Although the exact mechanism by which HSP90 inhibitors interfere with leukaemia cell survival is not fully understood, we have also demonstrated that 17-AAG was able to induce apoptosis in primary AML cells, in correlation with HSP90 and activated AKT levels [16]. In MDS, we show that 17-AAG readily inhibits CD34+ and MNC survival in liquid culture, at least in samples from high grade MDS. Short-term exposure to 17-AAG also down regulates pAKT and pFAK levels. This is consistent with the mechanism of 17-AAG-induced apoptosis suggested by Nimmanapalli *et al*, which implicates a modulation of apoptotic proteins of the bcl-2 family downstream of AKT, Raf-1 and Src pathways [37]. FAK is a cytoplasmic protein tyrosine kinase localized to regions called focal adhesions. Many stimuli can induce activation of FAK, including integrins and growth factors. The major site of autophosphorylation, tyrosine 397, is a docking site for the SH2 domains of Src family proteins. Phosphorylated FAK binds and activates proteins forming the FAK complex, and facilitates the generation of downstream signals necessary to regulate cell functions like motility, survival and proliferation. Dysregulation of FAK could participate in the development of cancer, and abnormal activation of FAK has been described in AML [29]. Our data suggest that FAK, as a client of HSP90, could be indirectly targeted by HSP90 inhibition.

Finally, although the HSP90 inhibitor alvespimycin proved to be toxic in patients with AML involved in a phase I study, other therapeutic strategies using HSP90 inhibitors may be possible, specially with new inhibitors like NVP-AUY922, which seems to be less toxic than ansamycine derivaties [38, 39]. Epigenetic therapies are increasingly used in MDS. The histone deacetylase (HDAC) inhibitors show a clinical activity in high-grade MDS. Acetylation of HSP90 by exposure to HDAC-6 inhibitors results in an inhibition of its chaperone function [40]. The combination of an inhibitor of HDAC and 17-AAG is highly active *in vitro* against cells from chronic myeloid leukemia in blast crisis and AML cells harboring a *FLT-3* mutation [41]. HDAC-6 inhibitors, alone or in combination with another HSP90 inhibitor, may therefore represent a potential targeted therapy of high risk MDS with possible dual mechanism of action. Recent preclinical studies have tested bortezomib, a proteasome inhibitor, in MDS, but with limited effects [42]. Adjunction of revlimid to bortezomib seems of interest, but recent data showed that efficacity of bortezomib may be enhanced by HSP90 inhibition, thought induction of protein misfolding. It will be of interest to test this combination in MDS [43].

In conclusion, assessment of the level of HSP90 expression could be predictive of patient response and allow the selection of patients for which HSP90 inhibition would be most promising.

## DESIGN AND METHODS

### Patients

One hundred and seventy-seven patients with MDS (n=154) and chronic myelomonocytic leukaemia (CMML, n=23) at diagnosis were included in this study between January 2006 and October 2010. All patients gave an informed consent. Diagnosis was carried out according to WHO recommendations and confirmed by two separate observers [26]. As we included CMML cases, the FAB classification was also used to distinguish sub-groups [27]. Cytogenetic analysis was available for 152 cases. Detailed clinical and biological characteristics are given in Table 1.

### MDS/CMML and control cells

Cells were collected by bone marrow aspiration into heparin-containing vials. Mononuclear cells (MNC) were separated on a Ficoll gradient (Eurobio, Les Ulis, France), washed twice with phosphate-buffered saline (PBS, Sigma-Aldrich, St Louis, MO), resuspended in RPMI 1640 (Eurobio) and incubated for two hours at 37°C on sterile plastic dishes. Non-adherent cells were then recovered, washed twice in PBS and immediately processed for further studies.

As controls, normal marrow cells were harvested from 6 healthy bone marrow donors and processed identically.

### Cultures

#### Short-term liquid cultures

17-AAG was purchased from Sigma-Aldrich, diluted in DMSO and stored at –20°C before use. Non-adherent MNC and CD34+ cells were incubated in RPMI1640 at 37°C in fully humidified atmosphere with 5% CO_2_ in the presence of different concentrations of 17 AAG (2μM or 5μM), or dimethylsulfoxide (DMSO) alone for controls, for 24 hours. After these treatments, cells were washed in PBS and viable cells were enumerated using a trypan blue exclusion test.

All experiments were performed in triplicate.

#### Clonogenic assays

Normal and MDS/CMML MNC were incubated in triplicate in methylcellulose and growth factor-containing culture medium (STEMα.ID, STEMα, Saint-Clément-les-Places, France). In some experiments, cultures were performed in the presence of 17-AAG (or DMSO alone for controls) which was added into the culture medium to obtain the appropriate final concentration (2μM or 5μM). CFU-GM and BFU-E were scored after 14 days of incubation.

### Antigen expression and flow cytometry

The samples were surface-stained with CD45-PECy5 (clone J33, Beckman-Coulter France, Villepinte, France) and CD34-FITC (clone 8G12, BD Biosciences, San Jose, CA, USA) antibodies for 15 minutes at room temperature. Then, the cells were washed and fixed with 3.7% formaldehyde (Sigma-Aldrich) for 20 minutes. The staining of intracellular proteins was performed after permeabilization in 0.2% Triton X100 (Sigma-Aldrich) during 15 minutes at room temperature. The specific antibodies used for this study were: HSP90-phycoerytrin (PE)-conjugated (clone F8 SCL3-119 which recognizes both HSP90 alpha and beta isoforms, Santa Cruz Biotechnology, Santa Cruz, CA), FAK-PE (clone H-1, Santa Cruz), pFAK-PE (clone K73-480, BD Biosciences), pAKTS473-alexafluor (AF647)-conjugated (clone M-89-61, BD Biosciences), and AKTS473-AF647 (clone 55PKBa/Akt, BD Biosciences). Cells were incubated for 1 hour at room temperature, washed and re-suspended in PBS before analysis. The isotype controls used for the phospho-proteins were matched to the primary antibodies at identical concentrations [28]. Cell populations were gated according to CD45/side scatter (SSC) analysis.

Flow cytometry analysis was performed with a Becton-Dickinson FACS Canto II, using the DIVA software. At least 100000 events were analyzed. Results were expressed as mean fluorescence intensity ratios (MFIR) (ratio of stained sample/isotype control).

### Apoptosis

#### Annexin V staining

Untreated and drug-treated cells were incubated with Annexin-V fluorescein and propidium iodide (PI) in HEPES buffer (Dako, Glostrup, Denmark). After incubation for 15 minutes in the dark, cells were analyzed by flow cytometry. Live cells were determined by PI exclusion. Early apoptotic fraction was determined by annexin-V-positive and PI-negative staining.

#### Activated caspase-3 expression

Cytospins were also used to study the caspase-3 activation by Alkaline Phosphatase-Anti Alkaline Phosphatase-(APAAP) technique. We used an amplification combination of alkaline phosphatase (AP) and avidin-biotinyled enzyme complex (ABC) technique (Vectastain Universal mouse and rabbit kit, Vector Laboratories, Burlingame, CA). Cytospins were fixed for 90 seconds with acetone at room temperature. They were rehydrated in PBS for 5 minutes. Non-specific binding was blocked with horse serum during 20 minutes. Then slides were incubated for 30 minutes with polyclonal rabbit anti active caspase-3 clone (Cell Signaling, Beverly, MA), washed twice in PBS for 5 minutes and incubated with biotinyled horse anti-mouse or rabbit secondary antibody for 30 minutes, rinsed again for 5 minutes in PBS, and reincubated for 30 minutes with biotinyled alkaline phosphatase complex (ABC reagent). After an additional wash in PBS, slides were incubated with an appropriate enzyme substrate solution (Fast red) until optimal red granular reaction (20-30 minutes). Slides were rinsed respectively with PBS and distilled water before a counterstain nuclear step consisting of an incubation for 7 minutes with Meyer's Haematoxylin (Dako). After washing in distilled water, PBS, and distilled water again, slides were finally mounted in Fluorotech aqueous media (Valbiotech, Paris, France). Controls were performed by replacing the primary rabbit antibody by an irrelevant antibody of the same isotype. Slides were examined using X10 magnification by two observers. Cells were considered stained if any diffuse reddish cytoplasmic staining could be identified. A scale of three levels of red staining was used to assess intensity of staining: 0 for no staining, 1 for weak staining, 2 for strong staining and 3 for very strong staining. The percentage of positive cells (strong or very strong staining) was determined after counting 100 cells.

### Statistical analysis

Mann-Whitney (or Kruskall-Wallis) non-parametric tests were used to compare the means of two (or more) groups. Proportions were compared by Chi-square test (or Fisher's test when a group comprised less than 10 units). Correlations were performed using a Spearman rank correlation test.

Survival curves were plotted according to the Kaplan-Meir method. Survival duration of different groups was compared by the log-rank test. Multivariate analysis of survival was performed using a Cox regression model.

Statistical tests were computed by the IBM SPSS statistical software and data plots were performed using the Prim5 software.
